# Optimizing early diagnosis by integrating multiple classifiers for predicting brain stroke and critical diseases

**DOI:** 10.1038/s41598-024-80129-3

**Published:** 2024-11-18

**Authors:** Ravnoor Singh, Satinder Kaur, Gurpreet Singh, Mehakdeep Kaur, Parminder Kaur

**Affiliations:** 1 Department of Computer Engineering and Technology, Guru Nanak Dev University, Amritsar, India; 2 Department of Computer Science, Guru Nanak Dev University, Amritsar, India

**Keywords:** Stroke, Classification, Machine learning, Data pre-processing, Hard voting, Ensemble learning, Neuroscience, Learning and memory

## Abstract

Machine learning has gained attention in the medical field. Continuous efforts are being made to develop robust models for early prognosis purposes. The brain is the most pivotal organ in the human body. A brain stroke is generally caused by a blockage in the brain arteries. A brain stroke is one of the primary reasons for death. Therefore, early prediction of diseases like brain stroke, heart attack can significantly help in making decisions for doctors. The research study aims to find a robust and potential technique for the early prediction of brain stroke, Alzheimer’s, heart attack, cancer, Parkinson’s and potentially reducing the incidence of severe post complications of the mentioned diseases. By considering the five datasets as input, machine learning models have been trained for the research study. Early prediction of brain stroke has been done using eight individual classifiers along with 56 other models which are designed by merging the pairs of individual models using soft and hard voting for brain stroke and eight individual classifiers have been used for early prediction of heart attack, cancer, Alzheimer and Parkinson’s. After analyzing the results of each classifier for each disease, the proposed method, which is a pair of random forest and decision tree using a hard voting method for early brain stroke prediction, achieves the highest accuracy of 99%, which is better than all classifiers. Along with accuracy, the proposed method attained a value of 98% in precision, an outstanding 100% in recall, and 99% in F1 score. XGBoost performed best for cancer, Parkinson’s, Alzeihmer’s and Bernoulli naive bayes performed best in case of Heart attack .Upon comparing the values of these performance metrics, they outshine all the other model’s values.

## Introduction

The stoppage in blood flow to the brain is the major cause of brain stroke, and strokes that occur because of this are called ischemic strokes^1^. The other type is hemorrhagic stroke, which is an effect of internal brain bleeding. Brain stroke can have short-term and long-term effects, depending on the quality of treatment. However, it can be a life-threatening event if not addressed timely. Strokes can affect the movement and functioning of the body. It can have severe effects, like paralysis or mobility issues^2^. Moreover, visual impairments, memory loss, and loss of senses are some of the reactions to a brain stroke. According to the WHO, annually, 15 million people all over the world experience a stroke. Among these 15 million people, 5 million patients die, and another 5 million people have to suffer from being permanently disabled^3^. People may suffer from strokes for various reasons, like diet, alcohol, tobacco, and medical history, but the most common ones include unhealthy and sedentary lifestyles. An unhealthy lifestyle may include the consumption of tobacco or other drug uses, whereas a sedentary lifestyle could lead to high blood pressure. According to WHO, the most significant modifiable risks are high blood pressure and tobacco use^3^. For every 10 people who died of stroke, four could have been saved if their blood pressure had been controlled. Two-fifths of the deaths from stroke under the age of 65 are associated with smoking. The only way to remove this menace is to reciprocate an unhealthy and lazy lifestyle. To curb the consequences of stroke, early prediction can help to a large extent. Even after advances in medical technology, the early diagnosis and prevention of strokes remain a major challenge. Traditional diagnostic methods often fail to identify high-risk individuals in a timely manner, leading to delayed treatment and poorer patient outcomes. The need for a reliable and efficient predictive model for strokes is critical to address this gap in medical care. Nowadays, artificial intelligence along with machine learning is becoming prevalent in the medical domain. There are numerous diseases that can be prevented or can be diagnosed adequately if anticipated earlier. Machine learning is mostly used for making programs for prognosis purposes in medical services^4,10–13^. Since accurate and timely diagnosis of brain strokes is crucial for improving patient outcomes and reducing mortality rates, this research aims to develop a robust stroke prediction model. By incorporating the machine learning techniques into clinical practice, diagnosis experts can benefit from enhanced decision-making support, ultimately improving patient outcomes. The model can be integrated into EHR systems to automatically analyze patient data in real-time. Alerts and recommendations can be generated based on the model’s predictions, providing actionable insights to healthcare providers. Most of the prognostic systems anticipate the illness using different factors like age, gender, body mass index, etc. After analyzing the factors and the patient’s medical history, classification algorithms are employed for prediction.

Earlier, various techniques have been used to predict early strokes^4^ implemented machine learning models along with an Artificial neural network (ANN) to anticipate early stroke. They found the weighted voting technique best in terms of ROC curve^5^ examined CT-scanned images to predict hemorrhage. Their research includes performance analysis of SVM with established prognostication tools like SEDAN and HAT scores^6^ implemented a hybrid model to anticipate stroke. The hybrid model includes random forest regression to impute missing values before classification. Afterward, a deep neural network based technique was applied^7^ use machine learning-based models to predict the outcomes of acute stroke^8^ conducted a study on different factors to predict stroke with SVM on 350 samples^9^ inculcated artificial intelligence for prognostic purposes. They used the decision tree method for the feature selection process, and afterward, they employed a back propagation neural network for building a classification model to achieve 97.7% accuracy^10^ provided a study that included the usage of deep learning and compared some machine learning algorithms. They compared the results of deep neural networks (DNN) with three machine learning techniques, which are gradient boosting decision trees, logistic regression, and support vector machines, and concluded that DNN achieves optimal results by using a lesser amount of patient data^11^ published research on three classification models to anticipate stroke and applied techniques to demographic data. The results of the decision tree were the most accurate, and Naive Bayes came out as the best in terms of the ROC curve^12^ implemented different classifiers like logistic regression (LR), decision tree (DT), random forest, and voting classifiers. Random forest was the best among all the implemented classifiers. ^13^ provided a performance comparison of the weighted voting technique with ten other machine learning classifiers. They concluded weighted voting was best based on accuracy, false positives, and false negatives^15^ examined different classifiers, namely Logistic Regression, Decision Tree Classification, Random Forest Classification, K-Nearest Neighbors, Support Vector Machine, and Naïve Bayes. They got the highest accuracy of 82% with Naive Bayes^17^ employed a hybrid deep feature engineering method for the classification of the brain images. ^18^ used a computer vision technique to classify a brain disease. Above mentioned studies examined various algorithms of machine learning and deep learning to anticipate strokes. However, among all the existing studies, the application of ensemble learning remains underexplored, which is a research void. This study includes a comparison of the machine learning algorithms with the new proposed methodology.

The paper proposes a hybrid model based on ensemble learning for predicting stroke using attributes such as age, gender, hypertension, body mass index level, heart disease, and smoking status. Performance comparisons of the proposed method with other classifiers like K-Neighbors classifier, random forest, SVM, Bernoulli Naive Bayes, decision tree classifier, XGBoost, Adaboost, and stochastic gradient descent have been done. Comparisons are done based on different metrics like accuracy, recall, precision, and false negatives. The proposed method can directly benefit patients by enhancing the medical decision-making process. Additionally, it will help society by lowering healthcare costs and increasing survival rates. The rest of the paper is organized as follows. Section 2 includes a description of the proposed methodology. Results and a comparison with existing work are done in Section 3. Section 4 sheds light on conclusions and future scope.

## Materials and methods

To accomplish the aim of the work, the research methodology is described in three main parts. The first part includes a description of the dataset. Afterward, machine learning classifiers used along with the proposed method have been described. The last part contains the procedure for implementation.

In early prediction model.

Let *X* represent the input variables or features used for early prediction.

Let *Y* represent the target variable indicating whether a person has disease risk or not.

The anticipation model can be represented as a function.

*f*(*X*) that maps input features to the target variable *Y*.

### Description of dataset

**Fig. 1:**
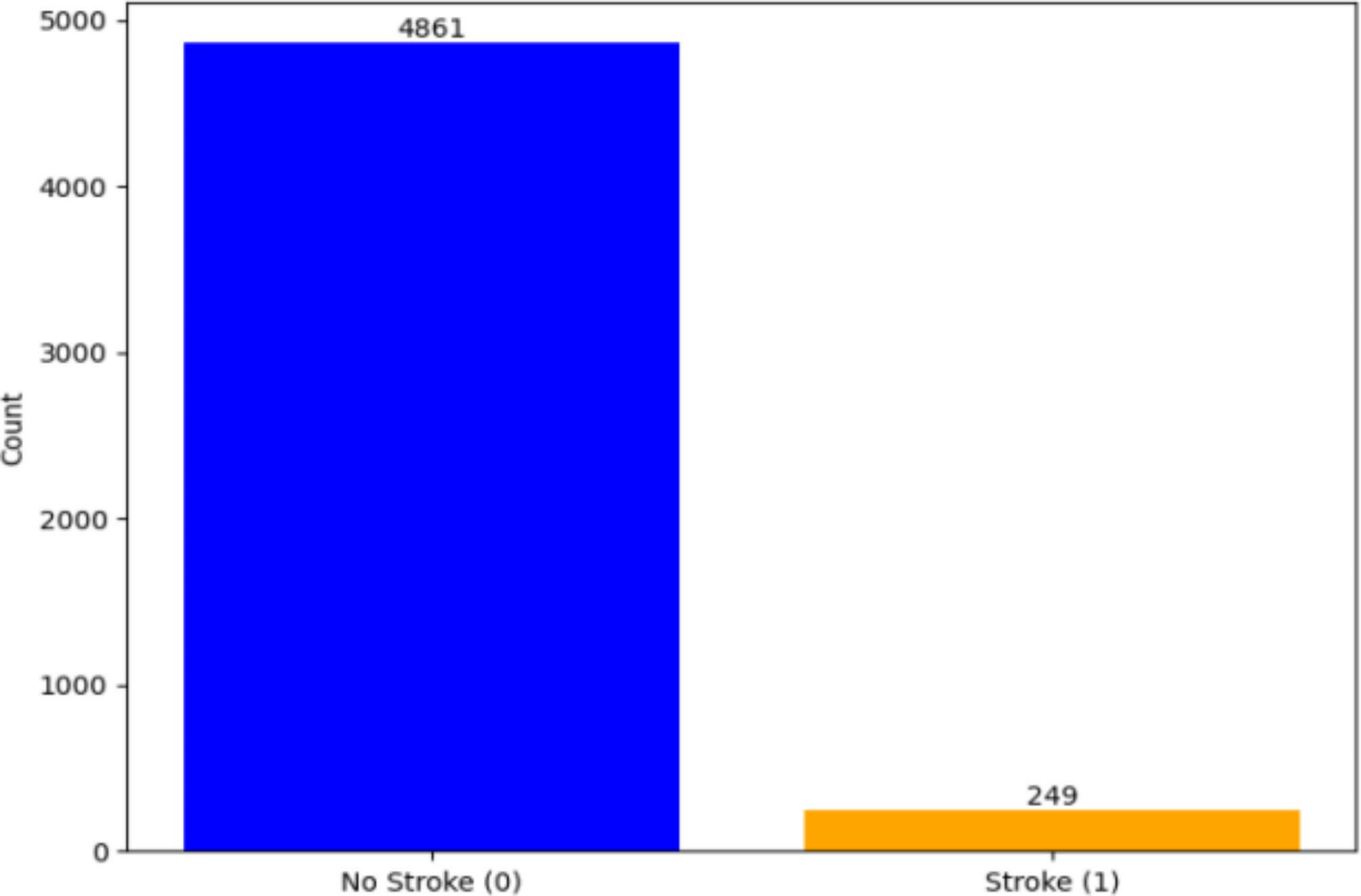
Total count of no stroke and stroke before data pre-processing.

The research was carried out using the stroke prediction dataset available on the Kaggle website. This dataset consists of 5110 rows and 12 columns. In the dataset, only 249 rows have a value of 1 for the stroke column, and the rest 4861 columns have a value of 0, as shown in Fig. 1. Hence, it is observed that the dataset is highly imbalanced. During the preprocessing phase, the dataset will be balanced.

Table 1 contains information related to the attributes of the dataset. In the table, the description column explains the feature and whether it is categorical data or numerical data is given in the data type column, The values of numerical data as well as categorical data are mentioned in the table. Among the 12 features, id is a unique identification number for each patient. Age is the second attribute and one of the major factors in determining stroke risk as the likelihood of experiencing a stroke increases with age. The dataset includes patients having an age range from 1 to 82 years offering a broad range of age to examine. The next attribute gender is another considerable feature as risk factors and stroke prevalence can vary significantly between males and females. Hypertension, a very common problem which occurs due to high blood pressure, is another important risk factor for brain stroke. This binary feature assists in identifying patients with hypertension, therefore aiding in the prediction. Another binary feature named heart_disease is also linked with stroke which helps in developing a more accurate predictive model. Ever_married describes the marital status of an individual. Marital status can influence stress levels and certain lifestyle choices which in turn affect health outcomes. The type of work can have a direct impact on both physical activity and stress level which eventually contribute in predicting brain stroke. The possible types of work included in the dataset are private, self-employed, govt_job, children, never_worked. The lifestyle and health facilities can be different in urban and rural regions which are relevant factors for brain stroke risk assessment. The type of region is given residence_type attribute. Average glucose level describes the level of sugar which further determines whether the patient is suffering from diabetes or not which is another important factor for early anticipation of stroke. BMI known as Body mass index is a measure of body fat based on height and weight. High BMI is associated with increased stroke risk due to its link with other health conditions. Smoking attribute describes the patient’s smoking habits. Smoking always bring diseases and it is a well documented risk factor stroke. The possible values of this attribute in the dataset are formerly smoked, never smoked, smokes and unknown. Stroke is the final attribute and target variable indicating stroke risk, with 0 for no stroke and 1 for stroke. This attribute is the primary outcome that the machine learning models aim to predict.

**Table 1:** Description of dataset.

Feature	Description	Data type	Values
ID	Unique identification number of patient	Numerical data	67, 68, 69…0.72940
Age (in years)	Age of patient	Numerical data	1, 2, 3…. 81,82
Gender	This attribute tells about gender	Categorical data	Male, Female
Hypertension	It tells whether the patient is hypertensive or not.	Numerical data	0,1
Heart_disease	This attribute gives information about heart disease	Numerical data	0,1
Ever_married	This attribute shows the marital status of the patient	Categorical data	Yes, No
Work_type	This feature gives information regarding the type of work	Categorical data	Private, Self-employed, Govt_job, children, Never_worked
Residence_type	It gives information regarding the residential area type	Categorical data	Urban, Rural
Average glucose level	It shows the average glucose level of the patient	Numerical data	55.12
BMI	This attribute denotes the body mass index	Numerical data	10.3, 16,17 .…0.97.6
Smoking_status	It represents the smoking condition of the patient	Categorical data	Formerly smoked, never smoked, smokes, unknown
Stroke	It indicates whether the patient has a stroke risk or not	Categorical data	0, 1

Table 2 shows the description of datasets used in for the experiments along with the number of tuples in the dataset. These datasets include different types of features. These datasets are a mixture of demographic features like ethnicity, age, residential type, educational level along with lifestyle and genetic features like smoking, alcohol, consumption, sleep quality, family history. The datasets have been processed and then divided into 70% and 30% ratios for training and testing purposes. Those datasets that contain some missing values have been filled using mean value and encoding of categorical data has been performed using one hot encoding.

**Table 2:** Experimental work datasets.

Dataset	No. of classes	No. of instances
Brain stroke	2	5110
Cancer	2	1500
Parkinson	2	2100
Heart attack	2	303
Alzheimer	2	2149

The last column name stroke can have either a value of 0 or 1. If the value is 0, then there is no brain stroke risk to the patient, whereas stroke risk was found if the value was 1. Figure 1 shows the huge difference between the two classes, which have stroke risk and no stroke risk. Among 5110 patients, only 249 have stroke risk, whereas the rest all do not have stroke risk.

**Fig. 2:**
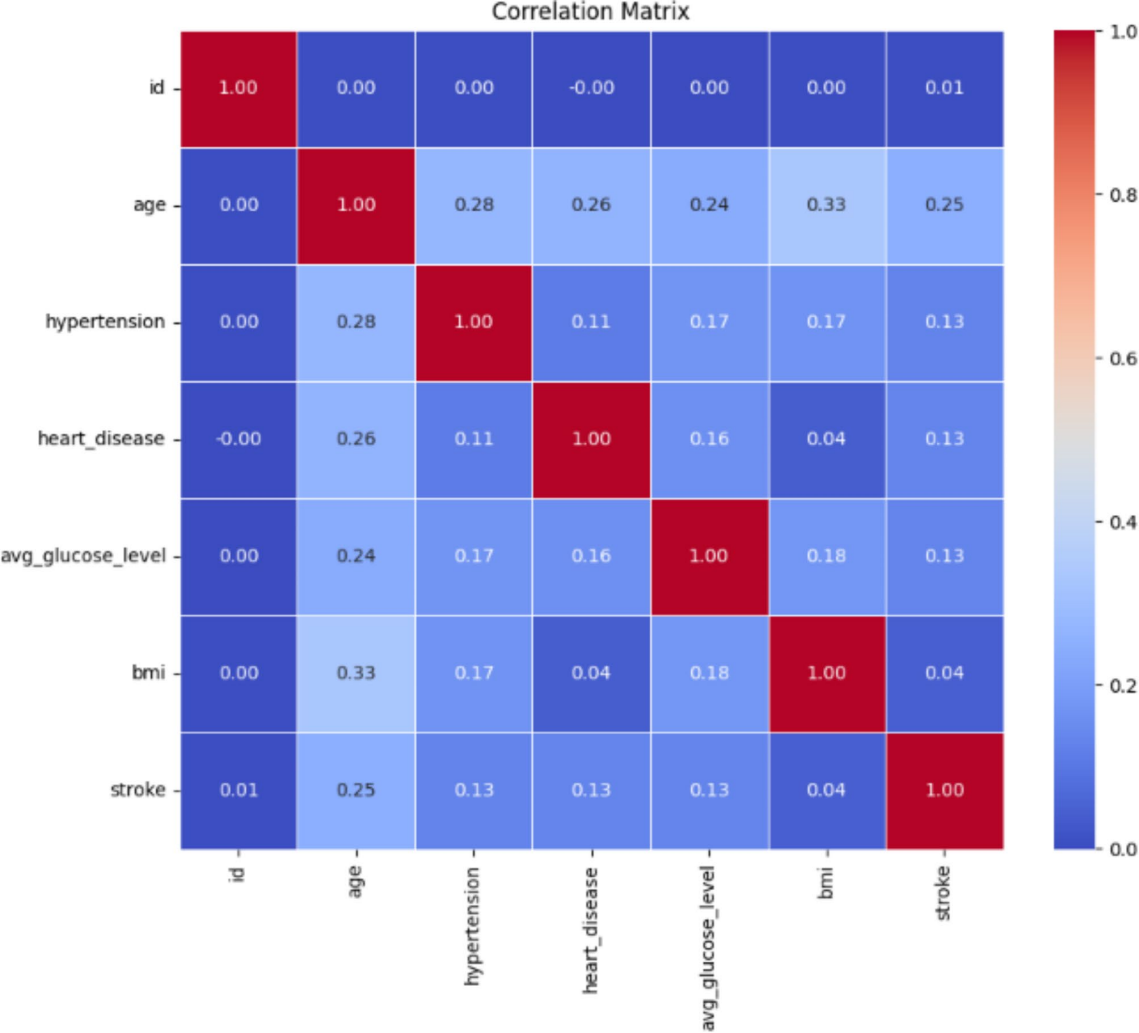
Correlation matrix.

**Fig. 3:**
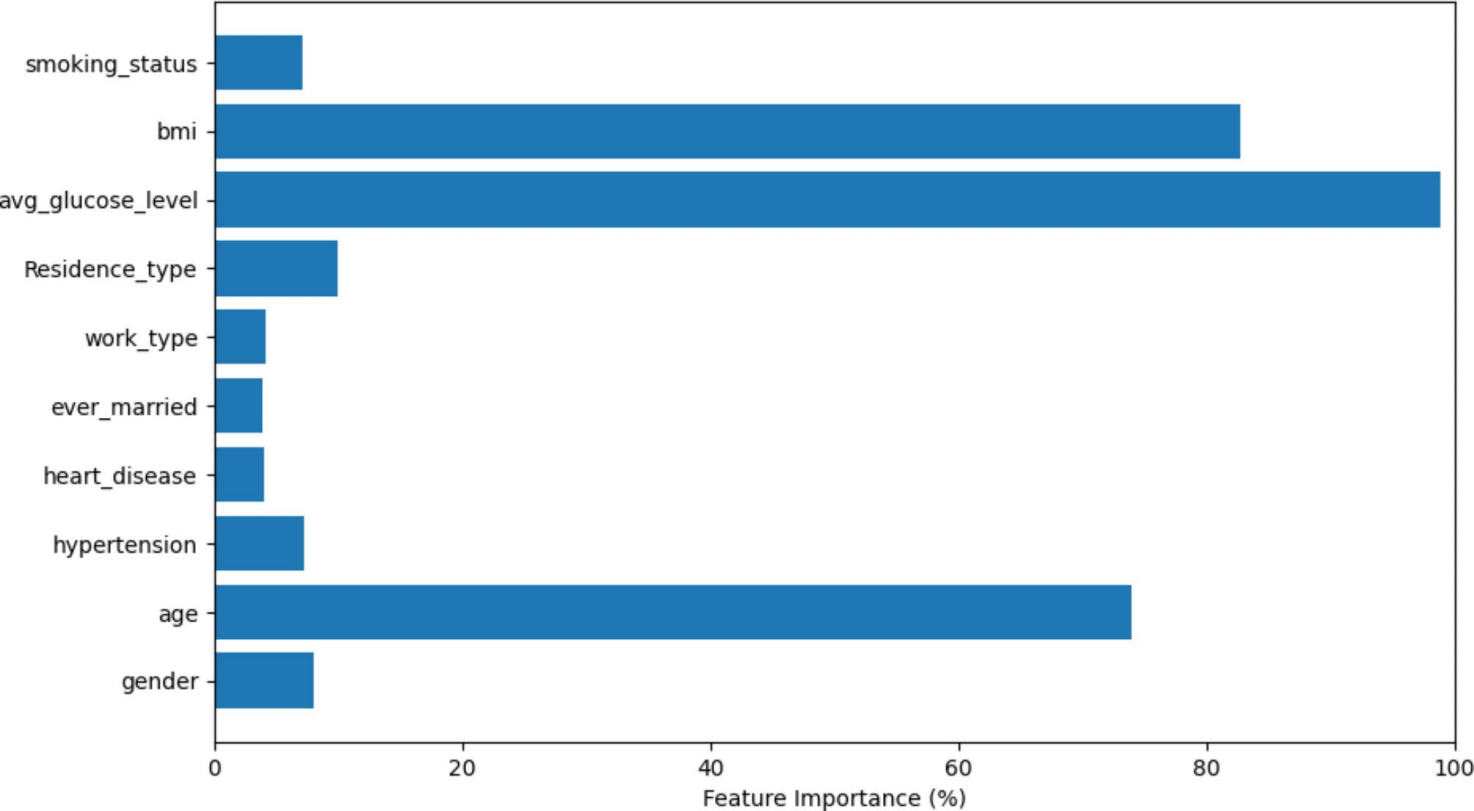
Feature importance.

Figure 2 shows the correlation matrix of numerical data. The correlation matrix provides an extensive overview of the relationships between the features of the dataset. In this matrix, each cell represents the correlation coefficient value, ranging from − 1 to 1. A positive value means a positive relationship. One as a value is considered a strong relationship, whereas 0 represents no relation. In Fig. 2, it can be observed that BMI (body mass index) is related to the age of the patient. It has been clear that the id being a collinear feature and not useful in anticipation, due to which the id column of each patient has been ignored during classification. A correlation matrix has been used to check for collinear data. Figure 3 represents the predictive power of the features used for brain stroke and contribution of each feature. The feature importance has been formulated by the light gradient boosting machine method. From Fig. 3, it can be observed that age, body mass index and average glucose level has the highest contribution.

### Machine learning classifiers

In this research study, eight individual classifiers have been used for classification of all diseases along with the 56 ensembled models for brain stroke. These 56 models have been developed using the combination of these eight classifiers into 2 pairs using hard and soft voting techniques. The selected models—Random Forest (RF), K-Nearest Neighbors (KNN), Support Vector Machine (SVM), Bernoulli Naive Bayes (BNB), Decision Tree (DT), XGBoost (XGB), AdaBoost (ADB), and Stochastic Gradient Descent (SGD) have been chosen for their diverse strengths and complementary features. RF, DT, and KNN are ensemble methods in themselves, offering robustness and reduced overfitting. SVM and SGD provide efficient handling of high-dimensional and large-scale data. Moreover, SVM prevents overfitting through regularization parameters and BNB is computationally efficient and very well-suited for binary feature classification. XGBoost and AdaBoost are advanced boosting algorithms known for their high performance and adaptability. Combining these models leverages their individual advantages, leading to a more accurate predictive system. Moreover, the further ensembling of these models has combined the strengths of the two models leading to more polished anticipation techniques. These classifiers have been used in several other research studies^11–13^. The proposed method based on ensemble learning is the blend of random forest and decision tree using a hard voting method. In the implementation of the proposed method, for the random forest, the value of parameter ‘n_estimators’ was set to 5 and ‘Gini impurity’ was set as a splitting criterion for the decision tree. This hard voting technique has been found to improve classification^14^.

### Implementation procedure

**Fig. 4:**
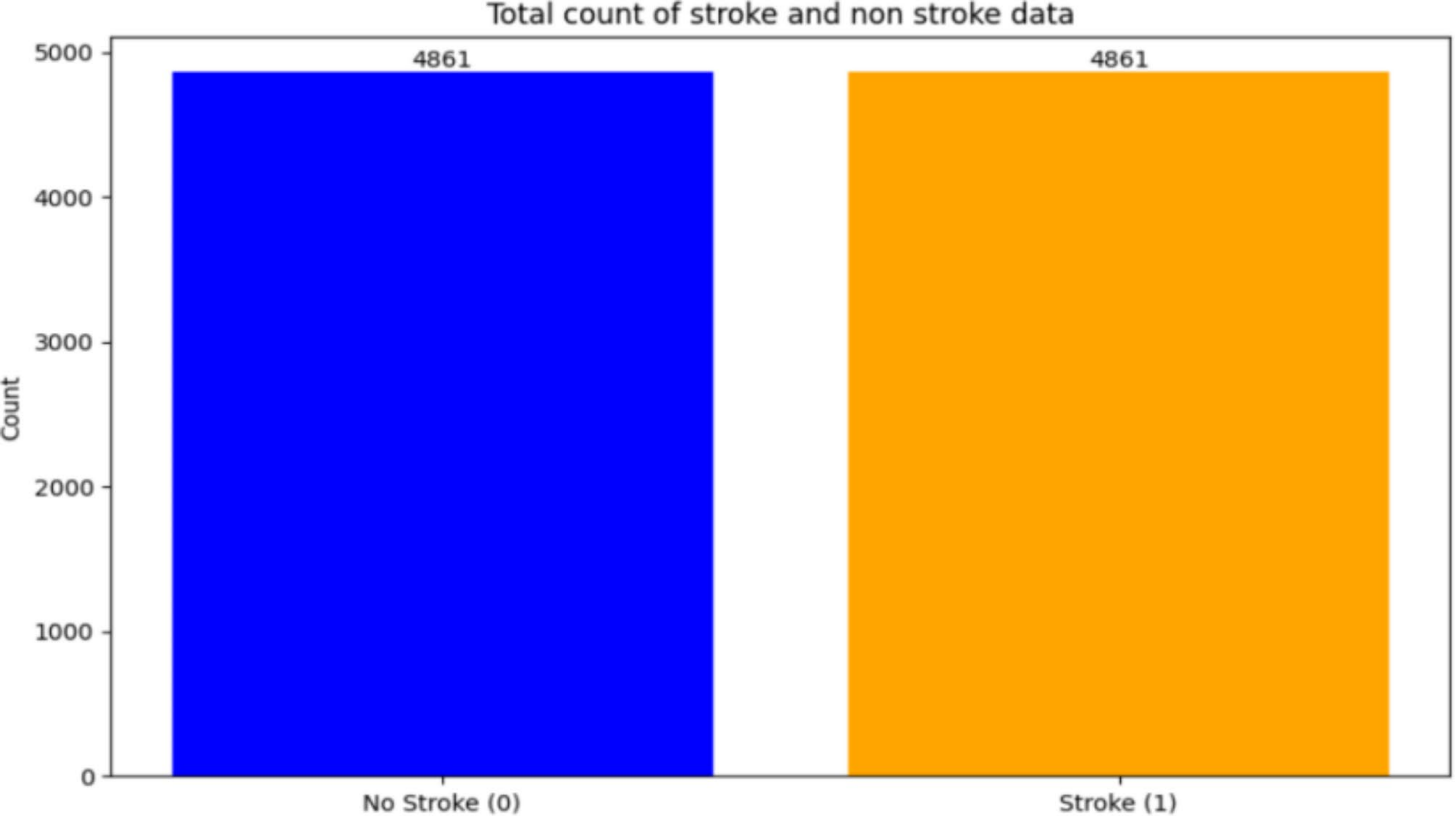
Total count of no stroke and stroke after random oversampling.

The main libraries used during implementation were Numpy, Pandas, Matplotlib, and Scikit-learn. Initially, in the implementation procedure, Data preprocessing is of utmost importance and it should be done before model construction in order to get the best results. In this step, raw data is processed to get converted into clean data. This stage deals with the issues that hinder the effectiveness of the model. As previously mentioned, the dataset of brain consists of 12 attributes. The first column of unique IDs has not been considered, as the ID hasn’t contributed to the prediction of stroke. All the datasets have been analyzed to identify whether there were any null or missing values and upon finding missing values, they have been filled. Among all the datasets, missing values has been spotted in the brain stroke dataset only. In the brain stroke dataset, the BMI column contains some missing values which could have been filled using either the median or mean of the column. Upon comparing the results, the models predicted more accurately when the missing values were filled by the mean of that feature using SimpleImputer. Thereafter, the categorical data was converted into numerical form. Two methods of encoding were tried: label encoding and one-hot encoding. Label encoding was not effective because it assigned numerical values to different categories, causing the models to give undue importance to higher numerical values. In contrast, one-hot encoding produced better results by representing categories as binary vectors, thereby eliminating any ordinal relationship and improving model performance. Categorical data has not been found in all the datasets except for the brain stroke dataset. Five columns of brain stroke were encoded into numerical form. Those columns are Gender, Ever_married, Work_type, Residence_type, and Smoking_status. Performance metrics like recall and precision are sensitive to the highly imbalanced dataset, and these metrics may not provide an accurate assessment of classifier performance. Since the dataset used is highly unbalanced, as mentioned earlier, it becomes imperative to address this problem. To curb this menace, oversampling techniques need to be implemented. Synthetic Minority Oversampling Technique (smote) and random oversampling technique has been tested to balance the data. Both techniques attained similar results with random oversampling having a slight edge in providing better results. After oversampling, both the classes stroke and no-stroke have been equal to 4861. Figure 4 shows the occurrences of having stroke risk and no stroke risk after oversampling.

Thereafter, all the dataset have been separated into 2 parts after data preprocessing : training and testing data. The split ratio of 70/30 has been used. After splitting, various scaling methods were employed to ensure the data was appropriately normalized. Initially, standard scaling (standardization) and normalization techniques were applied. Standard scaling transformed the data to have a mean of 0 and a standard deviation of 1, whereas normalization aimed to adjust the feature vectors to a common scale. Albeit, none of the two scaling methods yielded optimal results due to which the min-max technique was implemented. Min-Max scaling effectively scaled the data to fit within the range of 0 to 1. The min-max approach significantly improved the performance of the models, indicating their suitability for the dataset at hand. In order to get better results and to overcome the issue of overfitting, hyperparameter tuning was done to find the best possible parameters for the classifiers. The classifiers have been trained with the training data, and model evaluation has been done with the testing data. To find the best model and to perform performance analysis, testing data and performance metrics like accuracy, precision, and recall have been used.

**Fig. 5:**
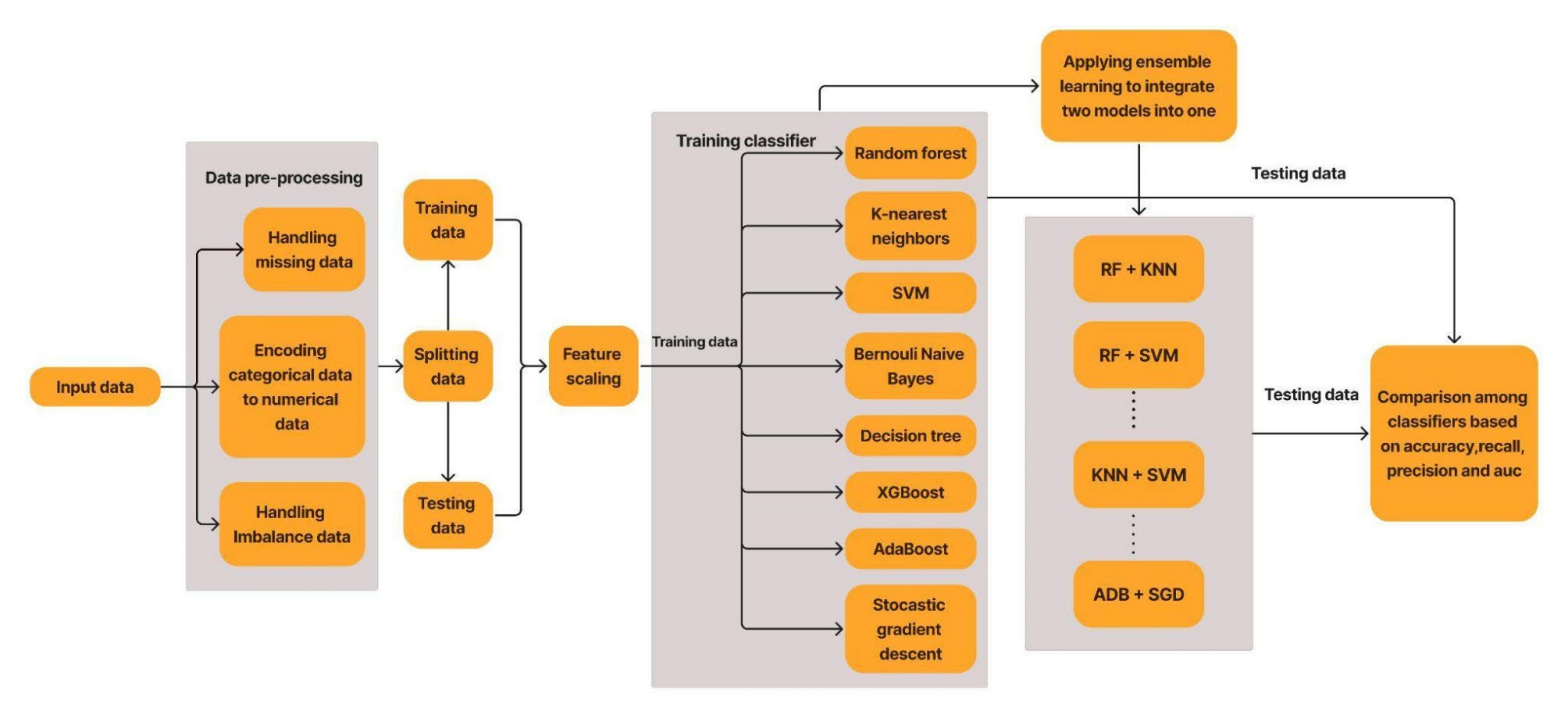
Flow chart of the procedure for early prediction.

Figure 5 depicts the implementation procedure for developing a classifier model. This flowchart has been designed using figma website^19^. The raw input data has been preprocessed, which includes encoding categorical data, handling missing data, and handling imbalance data. After data preprocessing, the data has been split into training and testing data. Feature scaling has been applied so that one large-scale feature does not dominate other features. Training and testing data have been used for the training and testing of models. In the end, the proposed method has been compared with all the other classifiers by using accuracy, recall, precision, and AUC as performance metrics for evaluation purposes.

## Results and discussions

The section uncovers the best-performing classifier. All the models have been evaluated based on confusion matrix, accuracy, recall and precision. The comparison of classifiers is important to find the best one. Different performance metrics check different aspects of the model’s performance. The accuracy tells the percentage of the correctly predicted values.

In classifier prediction:

- Let *C* represent the set of classifiers, which includes random forest, decision tree, and others.
- Each classifier *c* in *C* predicts the probability of stroke *P*(*Y* = 1∣*X*) given the input features *X*.

**Fig. 6:**
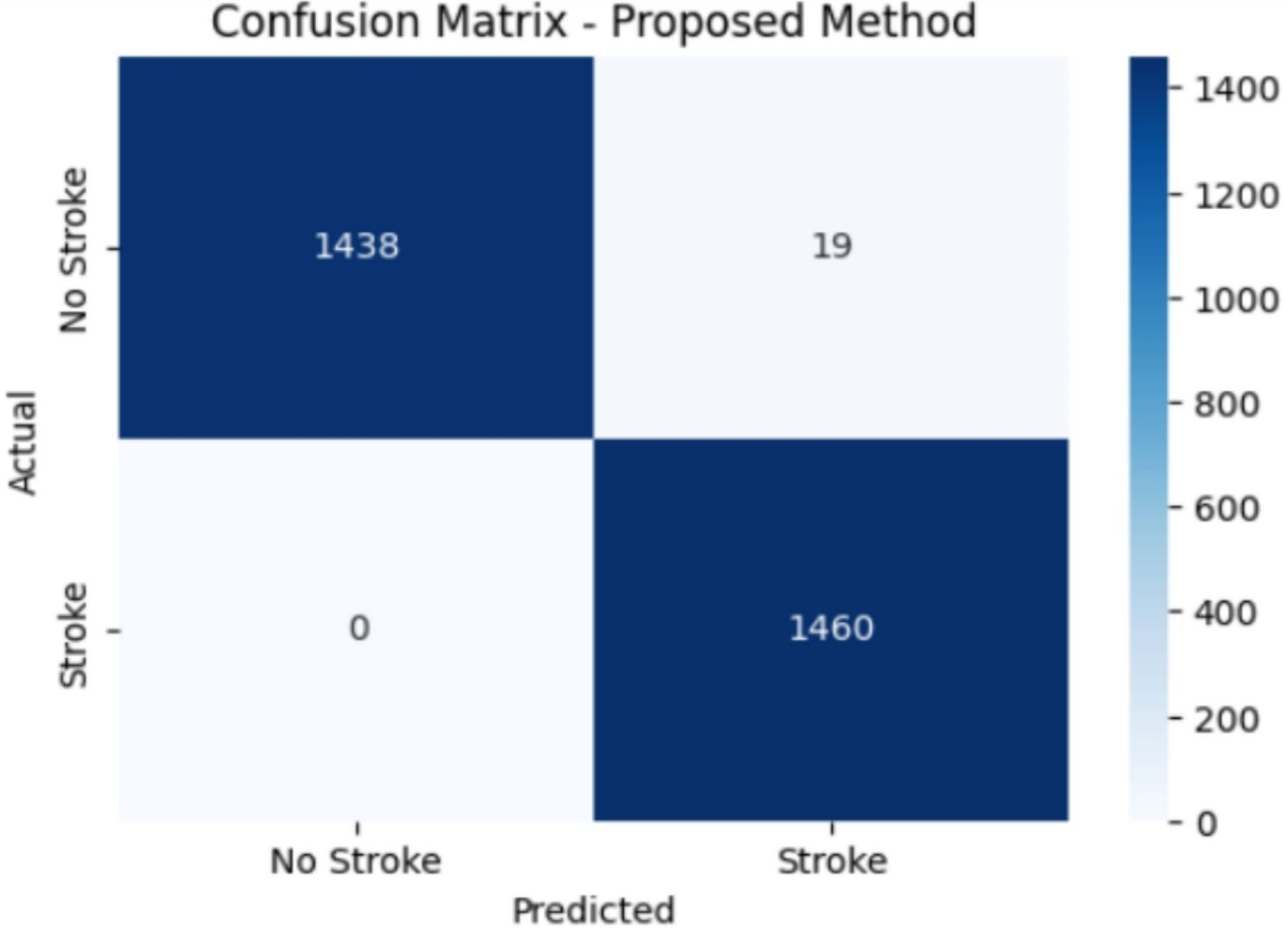
Confusion matrix of proposed method.

Figure 6 shows the confusion matrix of the proposed method. The confusion matrix allows the evaluation of different aspects of the classifier’s ability to predict. In Fig. 6, a comparison has been made between occurrences of predicted strokes and occurrences of actual strokes. From the confusion matrix, it can be observed that no patient is wrongly predicted as having no stroke risk, which is important in the medical field as it is dangerous for a patient to have stroke risk but predicted as having no stroke. It is very important to have the least possible false negatives as failing to identify patients having risk can lead to delayed or missed interventions and potentially worse outcomes .The confusion matrix helps in comparing the classifiers by identifying the true positive rate and false positive rate. Hence, the recall of this proposed method is 1. There are 19 patients who are wrongly predicted to have stroke risk. These Patients might undergo unnecessary tests, treatments, or hospitalizations, leading to increased healthcare costs and patient anxiety.

Table 3 displays six performance metrics of 64 classifiers used for evaluation purposes. In our study, we thoroughly assessed the effectiveness of these classifiers in predicting strokes. The table contains classifiers like K Nearest Neighbors, Random Forest, Support Vector Machine, Bernoulli Naive Bayes, Decision Tree, XGBoost, AdaBoost, and Stochastic Gradient Descent, as well as our proposed method along with other aggregated models using voting techniques. Each classifier was assessed based on metrics such as accuracy, precision, recall, F1 score, area under the curve, and K fold mean accuracy.

The mean accuracy in the K fold is computed by finding the mean of the accuracies of each fold. Five folds have been used in the K-fold cross-validation technique to check for overfitting. The K-nearest neighbors classifier got an F1-score of 90% and an AUC of 90%, with an accuracy of 90%, precision, and recall of 83% and 100%, respectively. With approximately 98% accuracy, favorable precision, and recall, the random forest and DT + XGB using hard voting achieved optimal performance, yielding an F1-score and an AUC of around 98%. In comparison to other classifiers, Support Vector Machine, Bernoulli Naive Bayes, and Stochastic Gradient Descent performed quite poorly, with accuracy rates ranging from 63 to 82% with variable precision, recall, and F1 scores. It can be observed that the model RF + XGB using hard voting achieved similar results compared to the proposed method however the proposed method outperformed all other classifiers in accuracy, precision, recall, F1 score, and AUC values with an accuracy of 99.30% and 99.22% in mean accuracy in stroke prediction. Table 3 presents comparisons that demonstrate the efficacy and potential of our proposed approach.

**Table 3:** Performance metrics of machine learning classifiers for brain stroke.

Classifiers name	Accuracy	Precision	Recall	F1-score	AUC	K-fold mean accuracy
K-nearest neighbors (KNN)	90.05%	83.42%	100%	90.96%	90.04%	91.46%
Random forest (RF)	98.01%	96.10%	100%	98.05%	98.00%	98.16%
Support vector machine (SVM)	82.44%	78.69%	89.04%	83.54%	82.44%	76.16%
Bernoulli Naive Bayes (BNB)	63.79%	62.22%	70.41%	66.06%	63.79%	63.86%
Decision tree (DT)	97.56%	95.36%	100%	97.62%	97.56%	97.54%
XGBoost (XGB)	97.22%	94.74%	100%	97.30%	97.22%	96.71%
AdaBoost (ADB)	86.15%	83.33%	90.41%	86.72%	86.14%	83.17%
Stochastic gradient descent (SGD)	78.95%	74.79%	87.39%	80.60%	78.94%	71.48%
Proposed method (RF + DT using hard voting )	99.30%	98.71%	100%	99.35%	99.34%	99.22%
RF + DT using soft voting	98.28%	96.68%	100%	98.31%	98.28%	98.40%
RF + KNN using hard voting	98.69%	97.46%	100%	98.71%	98.69%	98.89%
RF + KNN using soft voting	95.40%	91.59%	100%	95.61%	95.40%	96.12%
RF + SVM using hard voting	92.35%	96.60%	87.80%	91.99%	92.35%	89.28%
RF + SVM using soft voting	95.40%	91.59%	100%	95.61%	95.40%	94.95%
RF + BNB using hard voting	83.88%	96.43%	70.41%	81.39%	83.90%	83.00%
RF + BNB using soft voting	95.23%	91.30%	100%	95.45%	95.22%	94.78%
RF + XGB using hard voting	99.14%	98.31%	100%	99.15%	99.14%	99.13%
RF + XGB using soft voting	97.94%	96.05%	100%	97.98%	97.94%	97.93%
RF + ADB using hard voting	93.89%	97.20%	90.41%	93.68%	93.90%	92.57%
RF + ADB using soft voting	97.90%	95.98%	100%	97.95%	97.90%	98.38%
RF + SGD using hard voting	91.70%	95.65%	87.39%	91.33%	91.70%	83.98%
RF + SGD using soft voting	94.61%	90.29%	100%	94.89%	94.61%	82.04%
KNN + SVM using hard voting	86.49%	85.58%	87.80%	86.68%	86.49%	84.56%
KNN + SVM using soft voting	90.26%	83.71%	100%	91.13%	90.25%	90.73%
KNN + BNB using hard voting	78.19%	83.44%	70.41%	76.37%	78.20%	78.49%
KNN + BNB using soft voting	89.68%	82.90%	100%	90.65%	89.67%	90.72%
KNN + DT using hard voting	98.52%	97.13%	100%	98.54%	98.52%	98.41%
KNN + DT using soft voting	98.28%	96.68%	100%	98.31%	98.28%	98.07%
KNN + XGB using hard voting	98.28%	96.68%	100%	98.31%	98.28%	97.75%
KNN + XGB using soft voting	94.82%	90.62%	100%	95.08%	94.81%	94.65%
KNN + ADB using hard voting	90.74%	89.98%	91.71%	90.84%	90.74%	88.88%
KNN + ADB using soft voting	90.94%	84.92%	99.5%	91.67%	90.94%	92.04%
KNN + SGD using hard voting	86.45%	85.61%	87.67%	86.63%	86.45%	79.89%
KNN + SGD using soft voting	89.78%	83.04%	100%	90.73%	89.77%	81.22%
SVM + BNB using hard voting	72.27%	77.96%	63.49%	69.98%	72.75%	69.61%
SVM + BNB using soft voting	79.29%	76.75%	84.10%	80.26%	79.28%	74.81%
SVM + DT using hard voting	92.86%	95.82%	89.65%	92.63%	92.87%	89.07%
SVM + DT using soft voting	97.53%	95.30%	100%	97.59%	97.52%	97.54%
SVM + XGB using hard voting	92.18%	94.44%	89.65%	91.98%	92.18%	88.02%
SVM + XGB using soft voting	94.58%	91.20%	98.69%	94.80%	94.57%	94.33%
SVM + ADB using hard voting	85.08%	84.37%	83.16%	85.25%	85.08%	79.90
SVM + ADB using soft voting	85.15%	82.03%	90.06%	85.86%	85.15%	78.59%
SVM + SGD using hard voting	80.73%	79.08%	83.63%	81.29%	80.73%	72.32%
SVM + SGD using soft voting	80.76%	78.28%	85.20%	81.60%	80.76%	71.59%
BNB + DT using hard voting	84.91%	95.69%	73.15%	82.91%	84.92%	82.57%
BNB + DT using soft voting	80.49%	77.38%	86.23%	81.56%	80.48%	74.79%
BNB + XGB using hard voting	81.83%	94.28%	67.80%	78.88%	81.84%	81.86%
BNB + XGB using soft voting	94.65%	90.90%	99.24%	94.89%	94.64%	94.62%
BNB + ADB using hard voting	75.38%	83.66%	63.15%	71.97%	75.39%	73.62%
BNB + ADB using soft voting	73.94%	74.40%	73.08%	77.73%	73.94%	73.52%
BNB + SGD using hard voting	70.68%	73.80%	64.24%	68.69%	70.69%	65.96%
BNB + SGD using soft voting	75.11%	70.59%	86.16%	77.60%	75.10%	71.46%
DT + XGB using hard voting	98.83%	97.72%	100%	98.84%	98.83%	98.87%
DT + XGB using soft voting	97.53%	95.30%	100%	97.59%	97.52%	97.54%
DT + ADB using hard voting	93.62%	96.42%	90.61%	93.43%	93.62%	92.27%
DT + ADB using soft voting	97.53%	95.30%	100%	97.59%	97.52%	97.54%
DT + SGD using hard voting	92.35%	95.57%	88.83%	92.08%	92.35%	83.60%
DT + SGD using soft voting	97.66%	95.54%	100%	97.72%	97.66%	87.06%
XGB + ADB using hard voting	92.52%	94.54%	90.27%	92.36%	92.52%	91.13%
XGB + ADB using soft voting	96.67%	93.77%	100%	96.78%	96.67%	96.63%
XGB + SGD using hard voting	91.15%	93.91%	88.83%	91.30	91.53%	82.74%
XGB + SGD using soft voting	92.86%	87.52%	100%	93.35%	92.86%	70.79%
ADB + SGD using hard voting	82.34%	80.90%	84.72%	82.77%	82.34%	75.76%
ADB + SGD using soft voting	79.49	74.21%	90.47%	81.54%	79.48%	71.51%

**Table 4:** Accuracy of eight classifiers for different disease.

Dataset	K-nearest Neighbors (KNN)	Random forest (RF)	Support vector machine (SVM)	Bernoulli Naive Bayes (BNB)	Decision tree (DT)	XGBoost (XGB)	AdaBoost (ADB)	Stochastic gradient descent (SGD)
Cancer	70.46%	88.16%	63.42%	78.09%	88.16%	92.57%	89.39%	79.85%
Parkinson	72.46%	83.06%	80.22%	71.67%	86.55%	92.56%	91.45%	80.06%
Heart attack	81.31%	79.12%	83.51%	87.91%	75.82%	83.51%	72.52%	80.21%
Alzheimer	73.02%	90.04%	88.12%	74.46%	94.48%	96.16%	95.44%	83.33%

**Fig. 7:**
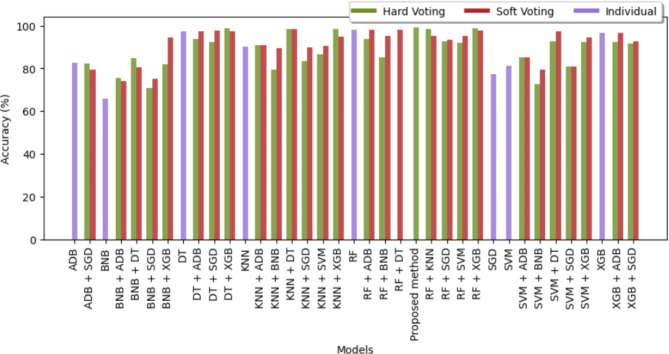
Comparison chart of accuracy of different classifiers for brain stroke.

**Fig. 8:**
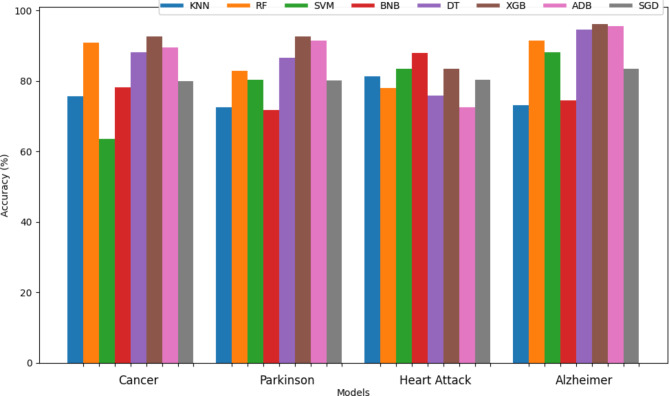
Comparison chart of accuracy of different classifiers for different classifiers.

Figures 7 and 8 are bar graphs that show the visual contrast between the accuracies of different algorithms that have been used in finding the best classifiers. Comparison in tabular form has also been done in Tables 3 and 4. Accuracy is the basic parameter for evaluating a classifier. Accuracy is calculated by dividing the number of correct predictions by the total number of predictions. It’s the percentage of correct classifications that a trained model achieves. The models combined through hard voting are shown in ‘olive-drab’ color and models developed by soft voting are shown in ‘firebrick’ color. For combinations of models, such as RF + KNN or RF + SVM, bars representing hard voting and soft voting are placed side by side without any space between them. This placement visually emphasizes the comparative performance of these voting strategies. Individual models are represented by a single bar centered on the x-axis label. The proposed method, which combines RF and DT using hard voting, is specifically labeled as “Proposed method”. From the graph, it has been observed that the proposed method (RF + DT through hard voting) has the highest accuracy, which demonstrates its potential for the prognosis of brain stroke. After the proposed method, the fused version of RF + XGB using hard voting attained 2nd highest accuracy of 99.14% followed by hard voting DT + XGB with an accuracy of 98.83%. XGBoost has performed best in classifying Cancer, Parkinson’s, Alzeihmer’s followed by ADAboost.

**Table 5:** Precision of eight classifiers for different disease.

Dataset	K-nearest neighbors (KNN)	Random forest (RF)	Support vector machine (SVM)	Bernoulli Naive Bayes (BNB)	Decision tree (DT)	XGBoost (XGB)	AdaBoost (ADB)	Stochastic gradient descent (SGD)
Cancer	75.61%	89.27%	63.80%	78.33%	88.73%	92.85%	89.00%	89.47%
Parkinson	75.78%	84.65%	80.32%	72.60%	86.82%	92.92%	92.36%	80.70%
Heart attack	73.58%	77.74%	75.47%	81.63%	71.73%	80.00%	66.66%	77.27%
Alzheimer	68.04%	89.95%	87.32%	76.02%	95.80%	95.50%	94.58%	83.90%

**Fig. 9:**
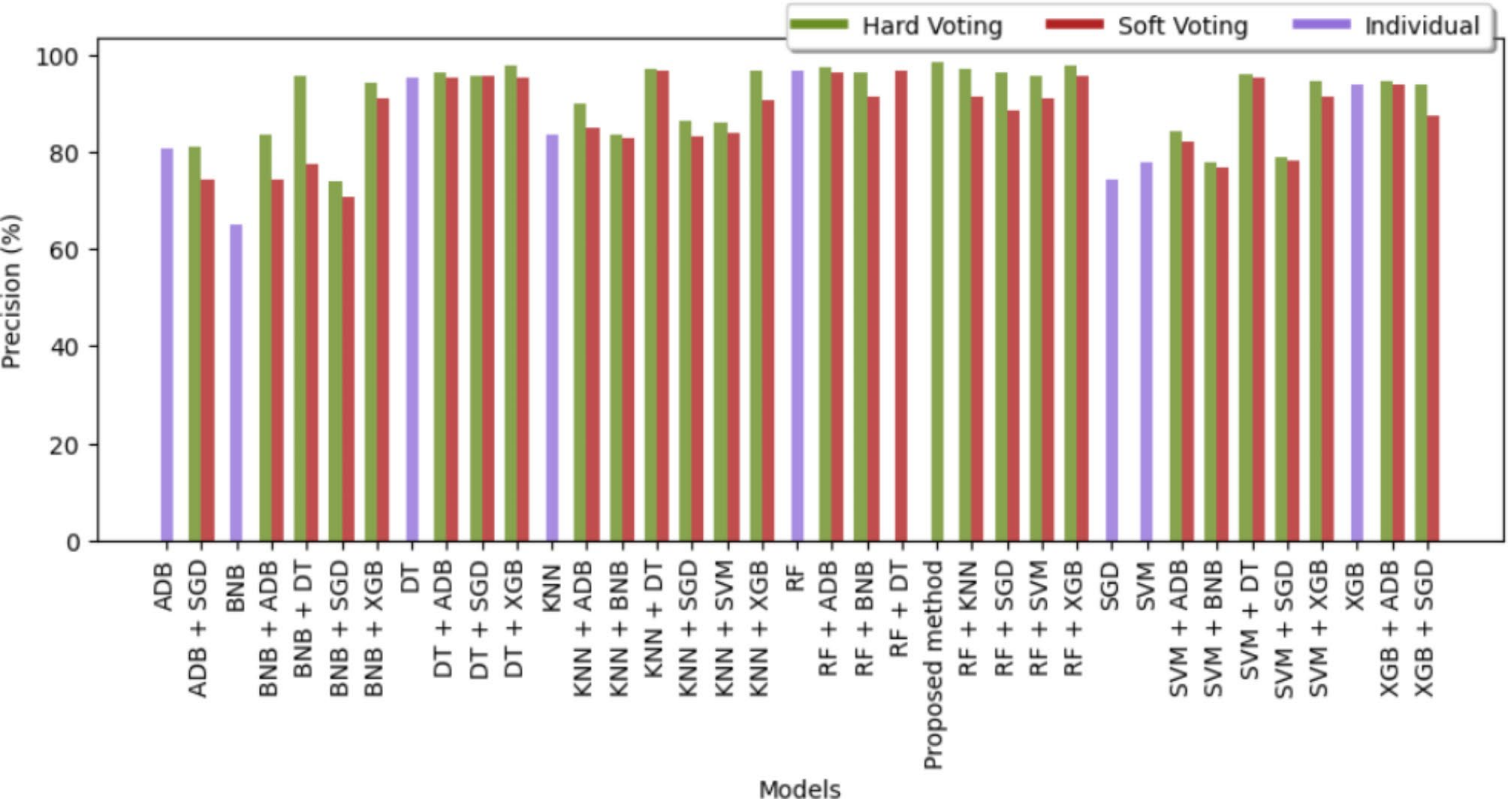
Precision of sixty-four different classifiers for anticipation of stroke.

**Fig. 10:**
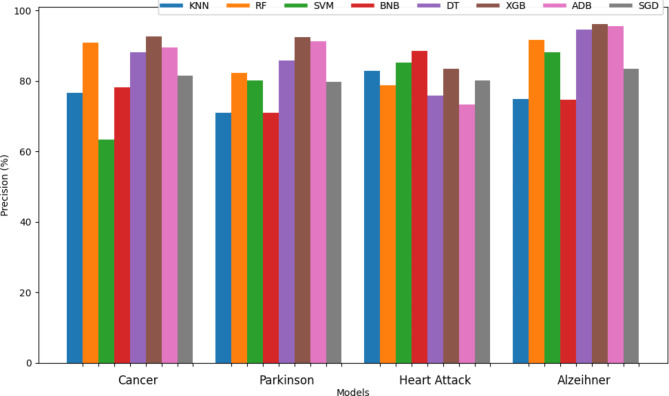
Precision of different classifiers for different diseases.

The precision of all the different classifiers is displayed in Table 5 for different diseases. The visual representation of the precision of different classifiers for brain stroke is shown in Fig. 9 and for the rest of the diseases is shown in Fig. 10. For the same combination of pairs like RF + SVM or DT + SGD, the bars of such models created by hard voting and soft voting are set together without any space between them. Among the individual models, it can be observed that only XGB, DT, and RF were able to attain optimal precision close to 95% whereas the precision of all the other individuals was below 90% and noticeably, BNB got the lowest precision of 62.22%. On the other hand, the majority of the classifiers designed using voting techniques are ahead by 90% which shows the aggregated strength of the two models. Along with the proposed method, only RF + XGB using hard voting was able to cross 98% for precision and DT + XGB using hard voting was on the edge of touching the mark of 98% precision. For Alzeihmer, XGB, ADB and DT attained similar precision as shown in Fig. 10. BNB came out to have the best precision among other classifiers in case of heart attack.

**Table 6:** Recall of eight classifiers for different disease.

Dataset	K-nearest neighbors (KNN)	Random forest (RF)	Support vector machine (SVM)	Bernoulli Naive Bayes (BNB)	Decision tree (DT)	XGBoost (XGB)	AdaBoost (ADB)	Stochastic gradient descent (SGD)
Cancer	67.00%	87.75%	68.36%	79.93%	88.43%	92.85%	90.81%	69.38%
Parkinson	88.87%	88.37%	89.66%	86.30%	86.21%	95.09%	93.79%	88.63%
Heart attack	92.85%	78.57%	95.23%	95.23%	78.57%	85.71%	80.95%	80.95%
Alzheimer	86.81%	90.16%	89.20%	71.46%	93.04%	96.88%	96.40%	82.49%

**Fig. 11:**
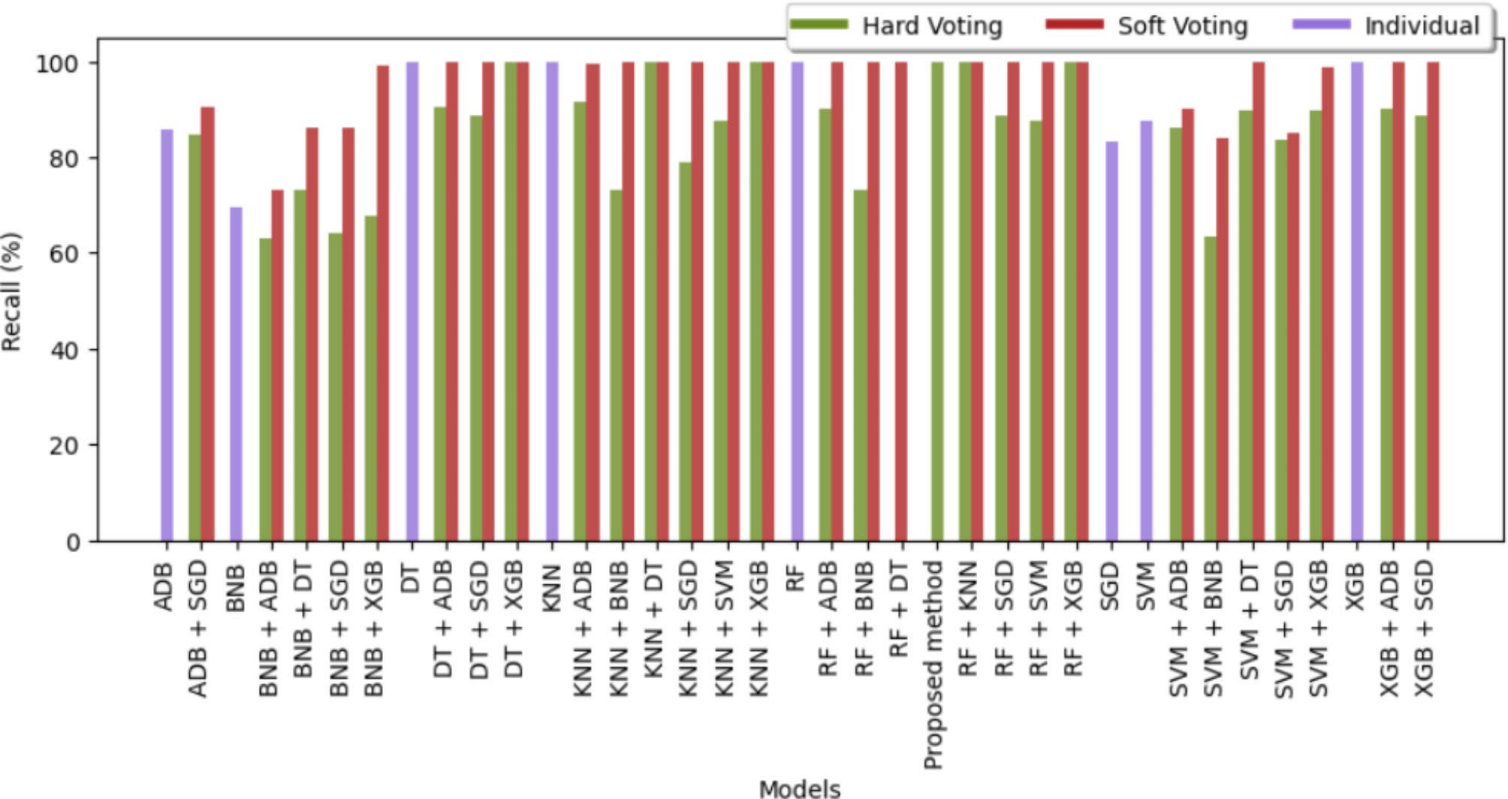
Visual representation of recall values of sixty-four different classifiers for brain stroke.

**Fig. 12:**
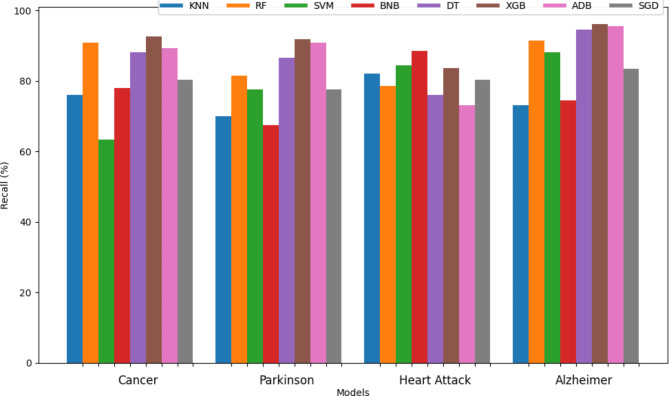
Visual representation of recall values of different classifiers.

The recall of different classifiers for all the diseases except brain stroke has been mentioned in the Table 6. Figures 11 and 12 represent the bar graph for comparison of recall also known as the true positive rate of all the sixty-four classifiers used. From the graph, it can be observed that the majority of classifiers developed by soft voting techniques were able to attain perfect recall whereas the number of hard voting models with perfect recall is less than soft voting models. Among the individual classifiers, RF, KNN, DT, and XGB achieved maximum recall whereas BNB attained only a recall of 70.41%. The SVM + BNB classifier designed by hard voting performed worst in terms of recall with a value of 63.49%. From Fig. 12, it can be observed that RF and BNB achieves highest recall for cancer and heart attack respectively however XGB consistently obtained optimal results for each disease.

**Table 7:** F1 score of eight classifiers for different disease.

Dataset	K-nearest neighbors (KNN)	Random forest (RF)	Support vector machine (SVM)	Bernoulli Naive Bayes (BNB)	Decision tree (DT)	XGBoost (XGB)	AdaBoost (ADB)	Stochastic gradient descent (SGD)
Cancer	74.06%	88.50%	66.00%	79.12%	88.58%	92.85%	89.89%	78.16%
Parkinson	78.24%	86.47%	84.73%	78.86%	88.77%	93.99%	93.07%	84.48%
Heart attack	92.85%	78.57%	95.23%	95.23%	78.57%	85.71%	80.95%	80.95%
Alzheimer	82.10%	77.64%	84.21%	87.91%	75.00%	82.75%	73.11%	79.06%

**Fig. 13:**
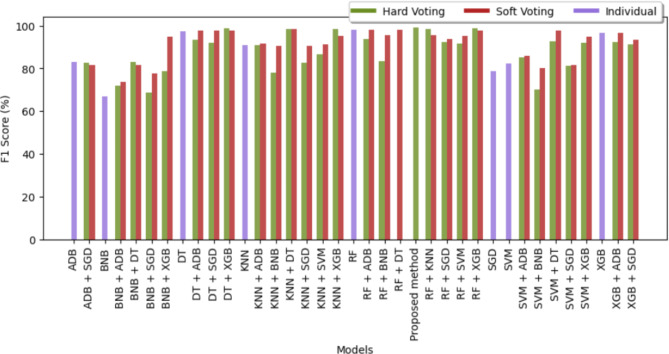
F1 score of sixty-four different classifiers for brain stroke.

**Fig. 14:**
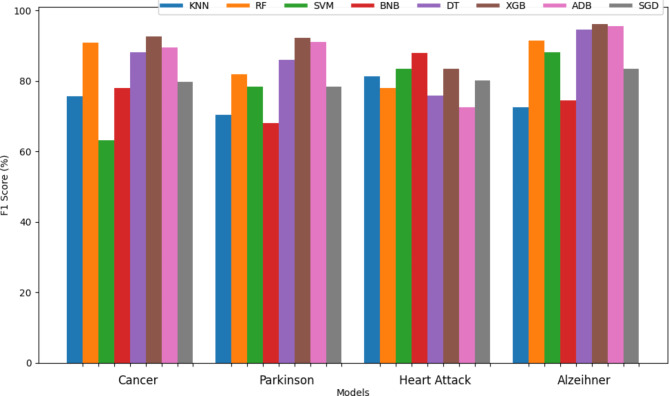
F1 score of sixty-four different classifiers for brain stroke.

The F1 score is the harmonic mean of recall and precision. Table 7 contains the values of the F1 score of the different classifiers for four diseases. SVM outperformed each classifier in case of heart attack meanwhile BNB got the best F1 score while anticipating Alzheimer. XGBoost got the best F1 score for Cancer and Parkinson’s. The graph of the f1 score of all the sixty-four classifiers is shown in Figs. 13 and 14 providing a comprehensive view of the performance metrics across various model combinations used in predicting strokes. Each bar represents a unique model pairing or individual classifier, differentiated by the voting strategy employed: hard, soft, or individual. From the graph, the combined models outperformed the individuals only RF, DT and XGB performed optimally in the case of f1 where multiple aggregated models performed better than these individuals. Only the proposed method and hard voting of RF + XGB were able to cross the mark of 99% whereas more than 8 models attained the f1 score greater than 98%. BNB was able to achieve only 66.06% which was the lowest among all the sixty-four classifiers for brain stroke.SVM got the lowest f1 score for cancer however it was able to attain second highest f1 score in case of heart attack.BNB got highest f1 score for heart attack and XGB got highest score for Cancer, Parkinson’s and Alzeihmer’s.

The results of the proposed method have been compared with the other studies and presented in Table 8. Each work is depicted with methods used for classification along with accuracy for comparative analysis of classifiers. The support vector^8^ has an optimal accuracy of 90%, whereas back propagation neural network^9^ achieves a higher accuracy of 97.7%, which indicates its efficacy. The accuracy of deep neural networks, decision trees, and naive bayes was below 90%, reflecting moderate performance. The random forest and weighted voting techniques achieved commendable accuracy of 96% and 97%, respectively, showcasing their potential. The proposed method did the best among all in Table 4 with an accuracy of 99%, and both its performance and scalability since it targets multiple diseases (brain stroke, heart attack, cancer, Parkinson’s disease) has potential advantages over the other models. The model by^16^ is for classifying acute ischemic infarction using pre-trained CNN models, whereas the work of ^17^ only utilized a hybrid deep feature engineering method to classify brain labels compared to multiple stroke and non-stroke disease classifications which include prediction on heart attack, cancer in addition through data from Alzheimer’s images. This drop and hence the havoc of this method will bring out is evident which highlights what vital importance such a proposed model anticipates for it to be applied predicting early disease risk.

**Table 8:** Comparison with other research work.

Existing work	Method	Accuracy
8	Support vector machine	90%
9	Back propagation neural network	97.7%
10	Deep neural network	87.3%
11	Decision tree	75%
12	Random forest	96%
13	Weighted voting	97%
15	Naive Bayes	82%
Proposed Method	Random Forest + Decision Tree using hard voting	99.3%

## Conclusion and future scope

Stroke, heart attack and other diseases can have lethal effects if not treated accurately. The integration of machine learning for prognostic purposes can help in anticipating early disease risk, which can lead to better decision-making and treatment. This research includes testing of multiple classifiers along with the proposed method and finding the best classifier for different diseases. By using accuracy, confusion matrix, recall and precision as performance metrics, a comparison of all the classifiers has been done. The use of hard voting solves this problem and gives the proposed method as having 1 recall so that all single stage learning algorithms are combined correctly to certain categories (brain stroke) where it has been found providing an accuracy estimation of 99%. Xgboost performed best for cancer, Parkinson’s and Alzheimer where Bernoulli naive Bayes gave superior results than other classifiers for heart attack. Because this study does not involve any imaging data, future studies might combine other types of data like MRI or CT-imaging that may increase predictive performance. Additionally, future work can extend our study by adding more diseases and traits.

## Data Availability

The datasets generated and analyzed during the current study are available from the corresponding to the author on reasonable request.
